# Neglected Diseases in the News: A Content Analysis of Recent International Media Coverage Focussing on Leishmaniasis and Trypanosomiasis

**DOI:** 10.1371/journal.pntd.0000234

**Published:** 2008-05-14

**Authors:** Mangai Balasegaram, Sooria Balasegaram, Denis Malvy, Pascal Millet

**Affiliations:** 1 Centre René Labusquière EA 3677, Bases Thérapeutiques des Inflammations et des Infections, Université Bordeaux 2, Bordeaux, France; 2 Health Protection Agency, North East and Central London HPU, London, United Kingdom; The George Washington University, United States of America

## Abstract

**Background:**

Although the pharmaceutical industry's “neglect” of neglected tropical diseases (NTDs) has been investigated, no study evaluating media coverage of NTDs has been published. Poor media coverage exacerbates the neglect. This study aimed to investigate, describe, and analyse international media coverage of “neglected diseases” in general and three specific NTDs—African trypanosomiasis, leishmaniasis, and Chagas disease—from 1 January 2003 to 1 June 2007.

**Methods:**

Archives of 11 leading international, English-language media were searched. A content analysis was done, coding for media organisation, date, author, type of report, slant, themes, and “frames”. Semi-structured interviews with journalists and key informants were conducted for further insight.

**Principal Findings:**

Only 113 articles in a 53-month time period met the inclusion criteria, with no strong trends or increases in coverage. Overall, the BBC had the highest coverage with 20 results, followed by the *Financial Times* and *Agence France Presse.* CNN had the least coverage with one result. The term “neglected diseases” had good media currency and “sleeping sickness” was far more widely used than trypanosomiasis. The disease most covered was leishmaniasis and the least covered was Chagas. Academic researchers were most commonly quoted as a main source, while the World Health Organization (WHO) and pharmaceutical industry were the least quoted. Journalists generally agreed NTDs had not been adequately covered, but said a lack of real news development and the need to cater to domestic audiences were major obstacles for NTD reporting. All journalists said health agencies, particularly WHO, were not communicating adequately about the burden of NTDs.

**Conclusions:**

Public health agencies need to raise priority for NTD advocacy. Innovative strategies, such as reporting grants or creating a network of voices, may be needed.

## Author Summary

In recent years, there has been a flurry of activity to reverse the neglect that has characterised NTDs, mostly focussed on drug development. The drug gap may be explained by market failure, yet other forces also conspire to cause the neglect of NTDs. One problem is the low visibility of these diseases. By comparison, the high-profile “big three” infectious diseases of AIDS, tuberculosis, and malaria have received increased donor attention and funding with greater visibility. Efforts to remove the “neglect” from NTDs must involve raising their profile. This study, focussing on three of the most neglected diseases, aims to provide a context of the current media situation—the what, where, and why of NTD coverage—to support future advocacy work.

## Introduction

Roughly one in six people globally, mostly the very poor, suffer from one or more NTDs. [1] These diseases may not directly result in high mortality rates, yet cause much morbidity, suffering and poverty. [2] Despite this, NTDs are a low priority for the pharmaceutical industry, lacking safe and effective treatments; are overlooked by mainstream global health efforts, receiving little funding; and are ignored by the media, rarely making headlines. Even public health authorities have downplayed NTDs – often, they are not perceived as health burdens and do not require compulsory reporting. [2]

In recent years, there has been a surge of activity around NTDs. The Drugs for Neglected Diseases Initiative (DNDi), kickstarted by Médecins Sans Frontières (MSF), and the Institute of OneWorld Health (IOWH), were both set up to help spur development of drugs. Through public-private partnerships, new drug projects have flourished, with 63 ongoing by the end of 2004. [3]

The drug gap from market failure has been studied. A 2006 study found that in the past 30 years, only10 drugs were marketed for “most neglected diseases”; (this figure rises to 21 if malaria and tuberculosis drugs are included). [4] However, NTDs and the media have not been studied. News reportage has been described as a “significant background” to policy change. [5] The importance of media advocacy in pushing forward tobacco control objectives has been demonstrated in studies. [6] It is thus timely and appropriate that greater attention be given to NTD advocacy.

This study aims to investigate, describe and analyse international media coverage of “neglected diseases” in general and three specific NTDs – African trypanosomiasis, leishmaniasis and Chagas disease (also known as American trypanosomiasis) – between 01 January 2003 and 01 June 2007. These parasitic diseases were chosen as they are some of the most neglected diseases, affecting people in three continents. The study period was timed around a key DNDi NTD advocacy campaign to ascertain whether the campaign had influenced media coverage. The study aims to provide a context of the current media situation facing NTDs and help future advocacy work.

## Methods

Electronic databases of selected media were searched for articles with the general term “neglected diseases” or the three NTDs selected from 01 January 2003 – 01 June 2007, a period that covered two years before and after the DNDi 2005 campaign. The quantitative component of the study measured the number and nature of news articles and noted any trends and patterns. A qualitative analysis reviewed the focus and perspectives of articles by identifying themes and “frames” – how issues are presented. The analysis was supplemented by interviewing nine journalists and four key informants on their perspectives of NTDs, news priorities and obstacles to coverage.

The study was restricted to English language media in order to standardise the analysis and enable comprehensive coverage within time constraints. It was also restricted to print media, which is common in media coverage analyses, due to the difficulty of obtaining complete records of radio or television coverage. As print and television coverage is generally strongly correlated, this was not expected to strongly distort the findings. [7]

### Inclusion Criteria for Media Databases

The media selected were BBC online, CNN.com, the international news wire *Agence France Presse* (AFP), the American news magazine *Time*, the international news magazine *The Economist,* the international business paper *Financial Times*, two British newspapers – *The Guardian* and *Daily Telegraph –* and three American newspapers – *The New York Times, Washington Post* and *Los Angeles Times.* Their databases are available online, some with a fee for access.

This selection was made as they have:

1. International coverage – with international print editions, broadcasts, wire services or audiences. The newspapers selected, for example, sell stories to newspapers all over the world. The Los Angeles Times-Washington Post news wire has been described as the “world's leading supplemental wire service” in an American Journalism Review survey. AFP publishes in both English and French and although it trails Reuters and AP in terms of income, it in fact covers the widest geographical area among the agencies, located in 165 countries, particularly in Asia and francophone Africa.
2. Significant financial and political weight – which could help influence international health policy through reaching donors and policymakers.

### Inclusion Criteria for Articles

Articles were defined as focussing on NTDs in general or one of the three NTDs studied if they had:

- at least two mentions of any of the search keywords;
- more than one paragraph with one mention of the search terms.

The search terms included the term “neglected diseases”, medical names of the three diseases plus the names “sleeping sickness”, “*kala azar*” and “black fever” (a literal translation of “*kala azar*”), which has been used in the US media without mention of any other disease name. Articles with only one mention of the term “neglected diseases” (in one paragraph) were excluded from the analysis but recorded separately to note how many times this term was used.

### Coding Variables

The coding system used was adapted from methodological frameworks used in other content analyses, particularly to track tobacco coverage. [8] [9] Articles were categorized by disease and media organisation, to note what diseases were covered and where, with a “general” category for articles discussing more than one ND but none in particular. Articles were also identified by author (if available), date, type of report (such as editorial or feature) and slant of reporting (negative, neutral or positive to NTD advocacy objectives). Qualitative analysis involved identifying topics and the “framing” of issues. Frame analysis has been described as a “means of explaining the ways that dominant news discourses evolve and come to define… a problem”. [5]

### Interviews

Semi-structured interviews were performed with nine leading health journalists to gain insight into the findings and investigate factors influencing reporting. Journalists were chosen from leading media organisations such as BBC, CNN, Reuters, AFP and Associated Press. Leading global health journalists from the *Financial Times* (FT), the *Boston Globe* and *Washington Post* were also interviewed. One academic and three former journalists now working on advocacy for international health agencies (two formerly with WHO) were also interviewed.

## Results

**Figure 1:**
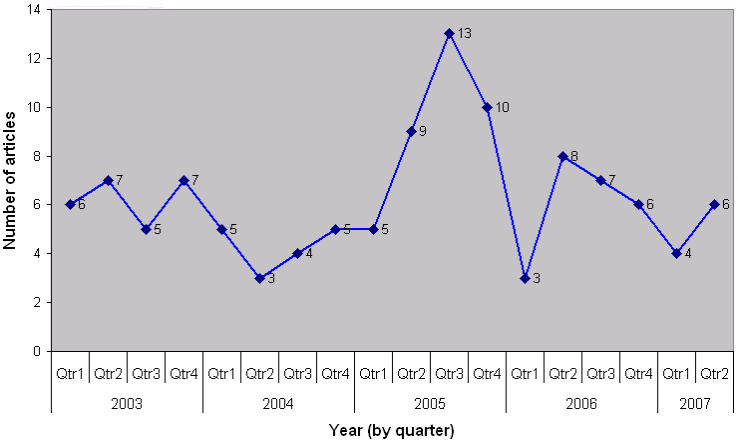
Count of articles over study period.

During the 53-month study period, 113 articles met the inclusion criteria. There were no strong trends or increases in coverage *(see* *Figure 1* *)*. A slight peak was noted in mid-2005 but there was no specific theme tied with this increase, although DNDi's research appeal campaign calling for greater attention to NTDs in May could have helped generate interest in this area.

Reporting on NTDs appeared to be sporadic and random, but a few events did prompt clusters of stories:

- a May 2003 NTD conference in Nairobi, Kenya;
- scientists “cracking” the genetic code of the three diseases studied;
- an 8 million pound grant to a UK university for tropical disease research;
- development of paromomycin for leishmaniasis treatment.

Most articles were hard news stories, with only one editorial and three letters (possibly indicating that NTDs do not evoke strong emotive responses). Reporting was generally sympathetic towards NTDs; a few articles were even critical of “Big Pharma” (*see Themes/Frames subsection)*.

### Media Organisations

The BBC had the highest coverage with 20 results, followed by the FT and AFP with 19 and 18 results respectively *(see* *Table 1* *)*. CNN had the least coverage with one result, for a story originally from Reuters. There were a wide variety of articles in the BBC and a notable number on sleeping sickness, including, uniquely, some from the field featuring patients. The FT had the most in-depth and detailed articles, often with exclusive financial perspectives. The new business model offered by public-private partnerships was explored in detail, in view of their potential for underfunded areas. The FT actually had the largest number of articles but many discussed the issue generally, rather in specific diseases, so did not meet the inclusion criteria.

**Table 1:** Articles found for each news organization.

Media Organisation	Total # Articles	General	Leishmaniasis	Sleeping sickness	Chagas
BBC (online)	20	8	3	8	1
Financial Times	19	17	0	2	0
Agence France Presse	18	7	6	5	0
The Guardian	15	8	5	2	0
New York Times	14	4	6	1	3
Washington Post	8	5	1	0	2
Los Angeles Times	8	2	2	1	3
Time Magazine	4	2	1	0	1
The Economist	3	3	0	0	0
Daily Telegraph	3	0	2	1	0
CNN.com	1	0	1	0	0
Grand Total	113	56	27	20	10

Although AFP's articles covered a wide area, reflecting the agency's mission, with three articles on sleeping sickness in Africa, none focussed on Chagas disease. Many stories from other organisations had a strong domestic angle, such as interviews with British scientists working on NTDs. The work of the US-based organisation IOWH, “black fever” among US troops in Iraq and the presence of Chagas in the US blood supply were some topics in the *New York Times.* The *Economist* offered long, in-depth analyses while none of the articles in *Time or Daily Telegraph* were “hard news” stories.

### Disease Category

The term “neglected diseases” was commonly used, which partly explained why many articles fell in the “general” category rather than a specific disease. The disease most covered was leishmaniasis, mainly because of the wide reach of those affected (which includes US troops in Iraq) and recent drug developments led by IOWH. African trypanosomiasis was the next most covered disease (as sleeping sickness), primarily by the BBC. Chagas had significantly less coverage with no articles in the British media other than one in the BBC. The main focus of Chagas by the American media was the parasite's threat to the American blood supply. No article actually focussed on the problem itself in South America.

### Sources Cited

The most common group to be quoted were local university researchers, accounting for main quotes in a third of articles with quotes. Academics represent a local, accessible and relatively independent source. With the “medical researchers” group, they accounted for 41% of all main quotes. WHO was quoted as a main source in only 4% of articles, while the Bill and Melinda Gates Foundation and pharmaceutical industry had even poorer media visibility. DNDi and MSF accounted for 18% of all main quotes.

### Themes/Frames

It was difficult to identify clear “frames”, but some broad themes of focus did emerge. The general need for more attention on NTDs, including calls for more research, drugs and funding, was the most common theme of articles collectively, with 19 articles. Public-private partnerships – which included the work of IOWH, DNDi and other such institutions - were another common focus, with 15 articles. Frames depicting the horror or tragedy of NTDs, often describing the reality of these “forgotten” diseases in term of the epidemic, victims, drugs or situation were almost as common. Other focuses for articles included:

- “Big Pharma”, where the industry was on the defensive or under pressure;
- scientific developments from genetic research;
- blood safety amidst the threat from Chagas (almost all US media articles).

### Interview Results

Journalists generally agreed that NTD were an important story that had not been adequately covered, but with the caveat that news stories had to be “newsworthy”. Health coverage veered towards “breaking news” such as bird flu outbreaks –headline-hitting events that raised ratings. Journalists who did cover NTDs were often personally motivated. Andrew Jack of the FT, who had the largest number of bylines in the study, said his reporting was “100%” driven by his interest.

A lack of real news development, the drive to cater to domestic audiences and competing health interests were cited as the main obstacles for NTD reporting. “Poor people dying from an illness is not news,” unless there is some change or development, one producer from an international broadcaster said. But HIV/AIDS was widely reported on “because it sells stories” and has the funding and attention of policymakers. Coverage of global health issues was particularly poor in the American media, where health and foreign budgets are facing cuts.

All journalists said health agencies were not communicating adequately about the burden of NTDs. Some journalists were particularly critical of WHO and the Bill and Melinda Gates Foundation for the difficulty in reaching officials for comment. Bill Gates was, however, credited with raising the profile of NTDs. NGOs such as MSF were cited as good sources for stories.

Journalists said stories needed to have a broad appeal which touched core readership to get covered. New developments or “breakthroughs” were easier to sell as stories. The “human element” was powerful, but few journalists were able to get such stories first-hand from the field. This represented a real constraint for coverage. One communications advisor (consulting for DNDi) said health agencies needed to present stories featuring “real people” rather than “experts in their ivory towers” and the “yuck” factor about these diseases needed to be played up to “grab the public imagination” rather than facts about the lifecycle of the parasite.

## Discussion

This study shows the general lack of coverage of NTDs in the media, with an average of about 10 articles per media organisation in a period of more than four years. By comparison, an unfiltered search for HIV/AIDS on AFP's database found more than 1,000 articles for the same period. There was a wide disparity in coverage between various media, with results for BBC 20-fold higher than CNN. No events or developments seemed to capture media interest across the board. The “newsworthy” element of NTDs clearly varied between media, ranging from the financial angles used by the FT to the emotive human stories featured in BBC.

The results reflect select international media – other leading organisations (such as Reuters news agency and the *Wall Street* Journal) had to be excluded due to time constraints. Also, only English-language media were selected and the term “international” is somewhat debatable. It would be useful to repeat the study in other language and compare ND coverage; a brief survey of *Le Monde* found many NTD articles, particularly on DNDi. Also, although some media had more profound articles on NTDs, this was not analysed due to a lack of an objective coding variable. However, this investigation is the first study to systematically analyse NTD media coverage. Further, the media selected still represent a sample of key media and some patterns clearly emerged. For example, the penchant for a local angle was even parochial at times. Stories get written about leishmaniasis in pets before humans, as was seen in The *Daily Telegraph.*

In the time frame of the study, activity by celebrities and the Global Network for NTDs (gnntdc.org), did not yet result in coverage by the mainstream media included in the study. However this may improve as celebrity activity and the networks pick up more media currency, especially with the impetus provided by President George Bush's 2008 NTD Initiative.

The interviews provided much insight, particularly on the struggle to cover global health issues in the American media, where foreign news budgets have been slashed. [10] One study found US foreign news coverage on front pages fell significantly from 1987 to 2004, from 27% to14%. [11] Interestingly, the news organisations with the first and third most coverage (BBC and AFP) both receive some public funding, so do not operate on an entirely commercial basis.

It is under such a challenging context that journalists face the pressure of reporting on relatively unknown diseases with limited information. Added to this is the difficulty in getting information on NTDs. Providing ready access to information and experts when needed is critical to improve coverage. Forming coalitions or networks could also help strengthen voices in the media.

In selling a story, terminology was enormously important. “Human African trypanosomiasis” was clearly off-putting for journalists, who overwhelmingly preferred the term “sleeping sickness”. Journalists also found “neglected diseases” more catchy and concise than “neglected tropical diseases”. Clearly, NTD advocates need to speak the same language as journalists to engage the media. The study also showed the lack of vivid and powerful “human” stories from the field (very few stories quoted patients) which generally have media appeal. One solution would be for NGOs to sponsor journalists to join them in the field, but this may raise the thorny issue of independent reporting.

In the market-driven setting of today's media, more innovative strategies may be needed. The same commercial context that constrains drug development of NTDs also curbs global health reporting, particularly in the American media. Just as public-private partnerships have transformed the landscape of drug development, some public-private funding may be needed to bring insightful, in-depth reporting on NTDs from the field to the pages of Western newspapers. Many fellowships, grants and awards are already available to promote reporting in certain fields. The Kaiser Family Foundation supports HIV/AIDS reporting projects [12] and offers international health fellowships while Harvard University recently started Nieman fellowships in global health reporting, with a US$1 million grant from the Bill and Melinda Gates Foundation. [13]

This study showed that even in a select group of media, there are clear patterns in what diseases get covered, what topics, terms and sources are preferred, and in which media. The disparity in coverage between media reflects different news priorities and interests, yet also opens a door to potentially increasing coverage, particularly amidst growing interest in global health. Public health agencies need to consider sustained and innovative advocacy on NTDs. A variety of strategies may be needed, including those to shift current “frames” – media portrayal and perception of NTDs.
